# The mutational profile of immune surveillance genes in diagnostic and refractory/relapsed DLBCLs

**DOI:** 10.1186/s12885-021-08556-3

**Published:** 2021-07-18

**Authors:** Marijana Nesic, Mads Sønderkær, Rasmus Froberg Brøndum, Tarec Christoffer El-Galaly, Inge Søkilde Pedersen, Martin Bøgsted, Karen Dybkær

**Affiliations:** 1 Department of Hematology, Aalborg University Hospital, Sdr. Skovvej 15, 9000 Aalborg, Denmark; 2 Department of Clinical Medicine, Aalborg University, Sdr. Skovvej 15, 9000 Aalborg, Denmark; 3 Clinical Cancer Research Centre, Aalborg University Hospital, Aalborg, Denmark; 4 Department of Molecular Diagnostics, Aalborg, Denmark

**Keywords:** Diagnostic DLBCL, Refractory/relapsed DLBCL, Immune surveillance, MHC class I, MHC class II, Somatic mutations, Gene, Immunotherapy

## Abstract

**Background:**

Diffuse large B-cell lymphoma (DLBCL) is the most frequent lymphoid neoplasm among adults,and approximately 30–40% of patients will experience relapse while 5–10% will suffer from primary refractory disease caused by different mechanisms, including treatment-induced resistance. For refractory and relapsed DLBCL (rrDLBCL) patients, early detection and understanding of the mechanisms controlling treatment resistance are of great importance to guide therapy decisions. Here, we have focused on genetic variations in immune surveillance genes in diagnostic DLBCL (dDLBCL) and rrDLBCL patients to elaborate on the suitability of new promising immunotherapies.

**Methods:**

Biopsies from 30 dDLBCL patients who did not progress or relapse during follow up and 17 rrDLBCL patients with refractory disease or who relapsed during follow up were analyzed by whole-exome sequencing, including matched individual germline samples to include only somatic genetic variants in downstream analysis of a curated list of 58 genes involved in major immune surveillance pathways.

**Results:**

More than 70% of both dDLBCLs and rrDLBCLs harbored alterations in immune surveillance genes, but rrDLBCL tumor samples have a lower number of genes affected compared to dDLBCL tumor samples. Increased gene mutation frequencies in rrDLBCLs were observed in more than half of the affected immune surveillance genes than dDLBCLs.

**Conclusion:**

Genetic variants in the antigen-presenting genes affect a higher number of rrDLBCL patients supporting an important role for these genes in tumor progression and development of refractory disease and relapse.

**Supplementary Information:**

The online version contains supplementary material available at 10.1186/s12885-021-08556-3.

## Background

Diffuse large B-cell lymphoma (DLBCL) is the most common form of adult lymphoma representing 25–35% of non-Hodgkin’s lymphomas. DLBCL is characterized by a high degree of molecular heterogeneity and genetics impacting patient stratification and treatment response [1, 2]. Although first-line therapy consisting of rituximab, cyclophosphamide, doxorubicin, vincristine, and prednisone (R-CHOP), cures a substantial proportion of de novo DLBCL patients, 30–40% relapse (~ 25%) or experience primary refractory disease (~ 15%) [3, 4]. The outcome of B-cell cancer treatment can be affected by somatic mutations of the cancer cells (intrinsic resistance), treatment-selected resistant subpopulations (acquired resistance), and genotype of the individual patient (inherent resistance) [5]. Unfortunately for the patients with primary refractory disease or relapse after first-line treatment, standard salvage treatments combined with autologous stem cell transplantation have limited efficacy and cannot be offered to all patients due to comorbidities or performance status [4, 6]. For those patients, immunotherapy modalities like immune checkpoint blockade (ICB) with antibodies targeting CTLA-4 or PD-L1/PD-1 or chimeric antigen receptor-T-cell (CAR-T) targeting CD19 have been considered due to its ability to restore functional anti-tumor immunity [7–9]. However, the response in rrDLBCL treated with ICB was not as efficient as hoped, potentially because the recognition of malignant cells by the host immune surveillance system was diminished or immune suppression mechanisms were activated [10]. CAR-T treatment has shown efficacy in rrDLBCLs but is sometimes followed by severe adverse events such as cytokine release syndrome and neurological toxicity along with B-cell aplasia [11, 12]. In dDLBCL immune surveillance can also be prevented through decreased recognition by effector cells and altered immune suppression and exhaustion mechanisms [13]. Therefore, there is an urgent need to characterize the genetics and underlying immune surveillance evasion mechanisms in DLBCL.

Of significant importance for immune surveillance is recognition of tumor cells by T-cells that require antigen presentation in association with major histocompatibility complex class I and class II (MHC class I and MHC class II) molecules [14, 15]. Whereas the NK cells recognize and attack the tumor cells with downregulated MHC class I molecules playing an important role in the host’s immune surveillance [16]. In dDLBCL, loss of expression of MHC class I and II molecules frequently occurs, 40–60% and 20–40%, respectively, while the concurrent loss of both molecules is observed in approximately 20% of cases [17, 18]. Sequencing data revealed that genes involved in antigen presentation to T-cells, activation of NK- and T-cells, and T-cell inhibition are recurrently mutated in both dDLBCL and rrDLBCL samples, implying that low immunogenicity of malignant B-cells gives an advantage in escaping the host immune surveillance system [19–24]. Loss of expression of MHC molecules on the cell surface of DLBCL cells can occur through multiple mechanisms [17, 25]. Genetic variants, including loss of function mutations in genes related to MHC proteins’ expression (e.g., *TAP1, TAP2, HLA, CREBBP, CIITA*), lead to one of the major mechanisms of tumor immune surveillance evasion described to date [17, 25, 26].

A limited number of studies have investigated aspects of immune surveillance in dDLBCL and even less in rrDLBCLs. However, recent research examining the genomic profile of rrDLBCLs suggested that hiding from immune surveillance is an intrinsic-resistance mechanism to R-CHOP-like therapies [27]. Since rrDLBCLs have low long-lasting response rates towards various salvage treatments and only limited benefit from ICB treatment, we set out to improve the insight and understanding of mutational profiles of immune surveillance genes in dDLBCL and rrDLBCL.

## Materials and methods

### Clinical samples

Patients with DLBCL from the Department of Hematology, Aalborg University Hospital, Denmark (the AAUH cohort) with dDLBCLs (*n* = 30) and rrDLBCLs (*n* = 17) were included in the study. Patients with transformed DLBCL, primary mediastinal large B-cell lymphoma, primary central nervous system DLBCL or primary cutaneous DLBCL were excluded. Diagnostic patients who relapsed during the follow-up period were excluded. All diagnostic patients were treated with R-CHOP or R-CHOP-like immunochemotherapy after diagnostic biopsies were collected. Four diagnostic samples were included from rrDLBCL patients (*n* = 4 matching dDLBCL and rrDLBCL patients) for additional analysis.

### DNA extraction

DNA and RNA were purified as previously described [28] from homogenized biopsies using Qiagen’s All Prep DNA/RNA/miRNA Kit, following the manufacturer’s guidelines. DNA from saliva or healthy tissue was purified using: DNeasy Blood & Tissue Kit (Qiagen, Germantown, MD, USA) and PrepITL2P (DNA Genotek, Ottawa, Canada), respectively following the manufacturer’s guidelines.

### Gene expression analysis and cell-of-origin (COO) classification

Patients were classified as activated B-cell like (ABC), germinal center B-cell like (GCB), or unclassified (UC) using CEL files obtained from Affymetrix GeneChip Human Genome U133 Plus 2.0 microarrays and methods implemented in the web-based tool (https://hemaclass.aau.dk) providing one-by-one Robust Multichip Average (RMA) normalization of microarrays and subsequent risk stratification of DLBCL into cell-of-origin, as previously described [29, 30]. Furthermore, CEL files were used for differential gene expression with the R-package limma [31] and Cibersort [32] analysis, using R version 4.0.3. Before the statistical analysis, gene expression data were background corrected and normalized using the RMA algorithm implemented in the R-package affy [33]. Expression was summarized at the gene level using a Brainarray custom CDF for the Affymetrix Human Genome U133 Plus 2.0 GeneChip.

### Sequencing

Library preparation was performed as previously described [28] using either the Accel-NGS 2S Hyb DNA Library Kit (SWIFT Biosciences, San Francisco, CA, USA) or Twist Library Preparation EF Kit (TWIST Biosciences, San Francisco, CA, USA) [28]. For exome capture, either the Twist Human Core Exome Kit (TWIST Biosciences, San Francisco, CA, USA) or Clinical Research Exome V2 (Agilent, Santa Clara, CA, USA) were used and further sequenced by Illumina paired-end sequencing producing a minimum of 26 Gb and 18 Gb of raw sequence data for tumor DNA and normal DNA samples, respectively.

### Bioinformatics workflow

An in-house developed workflow was used for data processing and analysis [28]. Briefly, raw FASTQ files were quality trimmed and checked using trimgalore v0.4.3 [28]. BWA mem v0.7.12 was used to align reads against the GDC GRCh38.d1.vd1 human reference genome sequence [28]. Further, somatic variants were detected using a combination of Mutect2 v3.8 and Varscan v2.4.1 [34, 35] and filtered using a minimum quality score (QSS) of 25, a minimum allele ratio (AF tumor/AF normal) of four, and a minimum allele frequency (AF) of 0.02. Variant annotation was performed by Ensembl’s variant effect predictor (VEP), annotating class, population allele frequencies, sift and polyphen predictions, genomic region, and protein domains [36]. The annotations from the cancer databases COSMIC Cancer Gene Census [37], OncoKB [22], CIViC [38], and ONgene [39] weres automatically assigned to each variant when applicable. To obtain high-quality data, filtering of variants was based on the following requirements: (1) that the minimum allele frequency of the altered allele is ≥5% and supported by ≥10 reads, (2) mapped in coding regions, (3) to be nonsynonymous or nonsense mutations, frameshift or indels and (4) to have high or moderate impact predicted by Ensembl variant effect predictor (VEP).

### Statistical analysis

Fisher’s exact test and Wilcoxon rank-sum test were used to compare groups of categorical and continuous variables, respectively. Hypothesis test with two-sided adjusted *P*-values < 0.05 were considered statistically significant. Bonferroni adjustment was used to account for multiple comparisons; *p*-values were adjusted for 78 tests. The statistical analysis was conducted using R (version 4.0.3) and GraphPad Prism (Version 7, GraphPad Software Inc., LaJolla, CA). For gene expression data analysis, *P*-values were adjusted according to the Benjamini-Hochberg procedure.

### Droplet digital PCR (ddPR)

The validation of selected variants was performed by ddPCR. Nonsense mutations with loss of protein function prediction in *CD58, TNRSF14*, and *CREBBP* genes were selected for validation. Positive controls (gBlocks Gene Fragments) purchased from Integrated DNA Technologies (IDT) were prepared by mixing with wild type (WT) gDNA from cell lines specific for each assay. Bio-Rad ID for assays and specific mutations are listed in Supplementary Table S1. The input of 66 ng purified gDNA (5 μL) was added to the reaction mixture of 11 μL of 1x ddPCR Supermix for Probes (No dUTP) (Bio-Rad), and 1x mutant target primers/probe (FAM)/ wild-type primers/probe (HEX) (1 μL) (Bio-Rad). Nuclease-free water was added, giving a total reaction mix volume of 22 μL. Emulsion droplets were generated by the QX200 Droplet Generator (Bio-Rad), following the transfer of droplets to a 96-well PCR plate. Two-step thermocycling protocol (95 °C × 10 min; 40 cycles of [94 °C × 30 s, 60 °C × 60 s (ramp rate set to 2 °C/s)], 98 °C × 10 min) was carried out in C1000 Touch Thermal Cycler with 96 Deep Well Reaction Modules (Bio-Rad). End-point fluorescence within each droplet was measured using QX200 Droplet Reader (Bio-Rad). Data were processed using the QuantaSoft Analysis Pro software program (Bio-Rad).

### External validation cohorts

For validation of dDLBCL, data from Chapuy et al., 2018 (135 dDLBCLs, which are a mix of cured and relapsing diagnostic samples) were utilized [40]. For refractory/relapsed DLBCL, the Morin et al., 2016 (25 rrDLBCL cases) and Greenawalt et al.,2017 (47 rrDLBCL cases) cohorts were used for validation [19, 23]. The validation cohorts are named by the author’s name. The validation datasets were publicly available as VCF files for all cohorts, which were filtered and analyzed in the same manner as our data.

## Results

### Clinical characteristics

The median follow-up for the included patients was 7.6 +/− 3.2 years from the time of diagnosis. Patient characteristics at the time of diagnosis for the 30 dDLBCL and 17 rrDLBCL patients are shown in Table 1. In rrDLBCLs, fifteen patients relapsed within the first two years from diagnosis, and the remaining two patients relapsed after 4.7 and 6 years from diagnosis. Only lactate dehydrogenase (LDH) differed significantly at the time of diagnosis between DLBCL patients that subsequently were cured and experienced refractory disease or relapse. At the time of diagnosis, the mean age was 64 years for both dDLBCL and rrDLBCL patients, ranging from 31 to 84 and 45–80, respectively. Molecular subclassification into ABC and GCB for dDLBCL patients resulted in 46% as ABC, 33% as GCB, and 20% as UC classified, and for17 rrDLBCL patients 65% were ABC, 24% were GCB, and 33% were UC classified (Table 2).

**Table 1:**
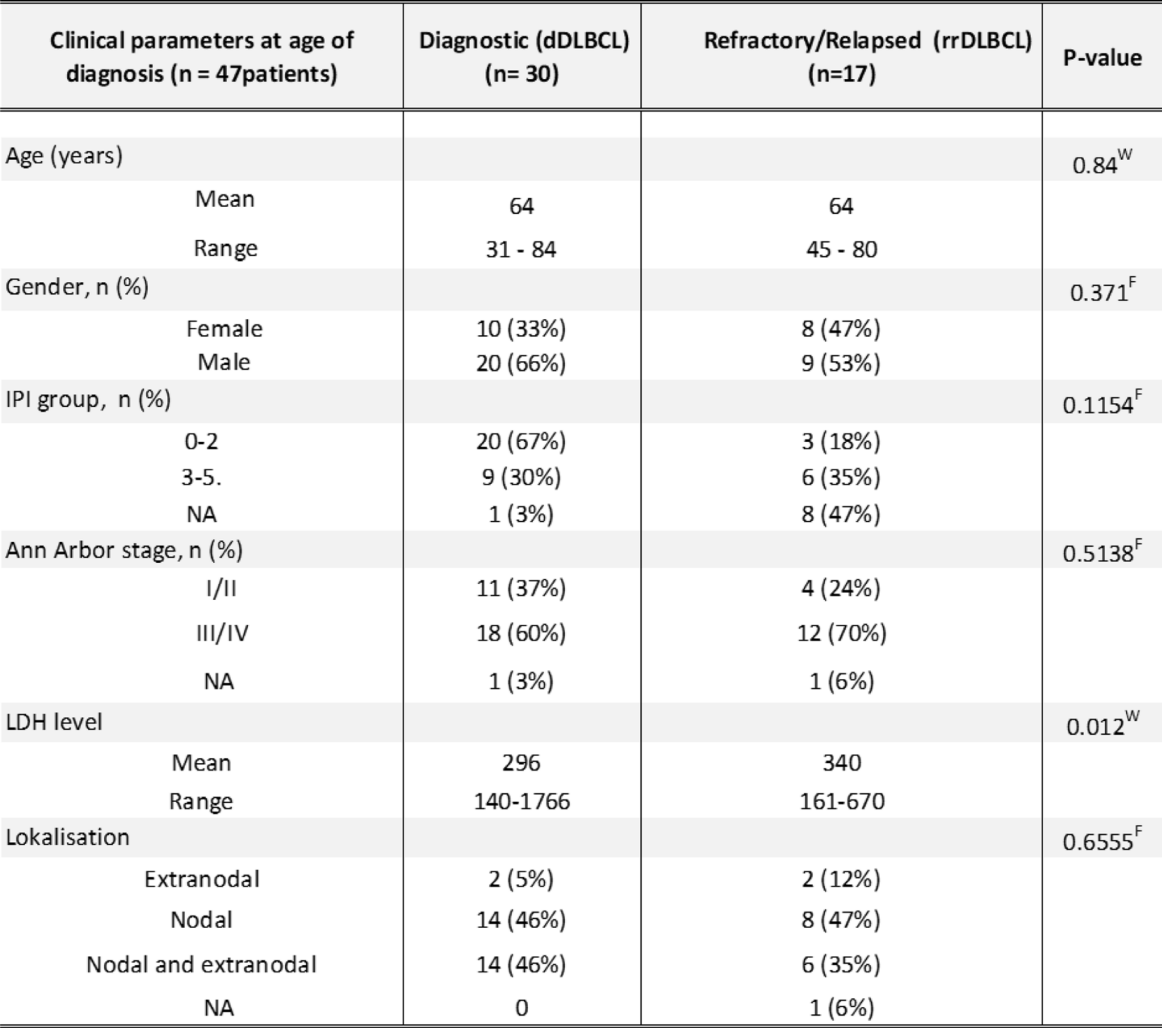
Clinical characteristics of the diagnostic and refractory/relapsed cohort. Clinical parameters at age of diagnosis

^F^Fisher’s exact test, ^W^Wilcoxon rank-sum test, n, number of patients; *NA* not available

**Table 2:**
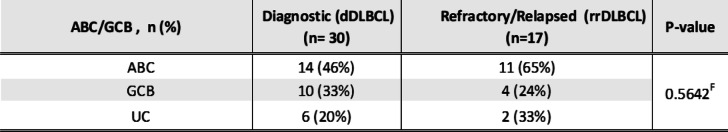
Clinical characteristics of the diagnostic and refractory/relapsed cohort. Cell-of-origin classification at the time of diagnosis and the relapse

^F^Fisher's exact test, n, number of patients

### Sequencing and gene set selection

Whole-exome sequencing (WES) was performed on 47 tumor samples with a matched germline sample, having a median of the sample mean coverages of 128× (range 47–331) and 97× (range 61–205), respectively.

From a curated list of 58 genes encompassing all major immune surveillance pathways [21, 41, 42], genetic variants were detected in 48 out of 58 genes in our dataset. A total of 242 somatic variants were detected in the 48 affected immune surveillance genes, which after filtering for quality, resulted in 147 nonsynonymous, nonsense, small frameshifts, and splice variants in a total of 36 immune surveillance genes included in downstream analysis.

### Mutational profile of immune surveillance genes in dDLBCLs and rrDLBCLs

**Fig. 1:**
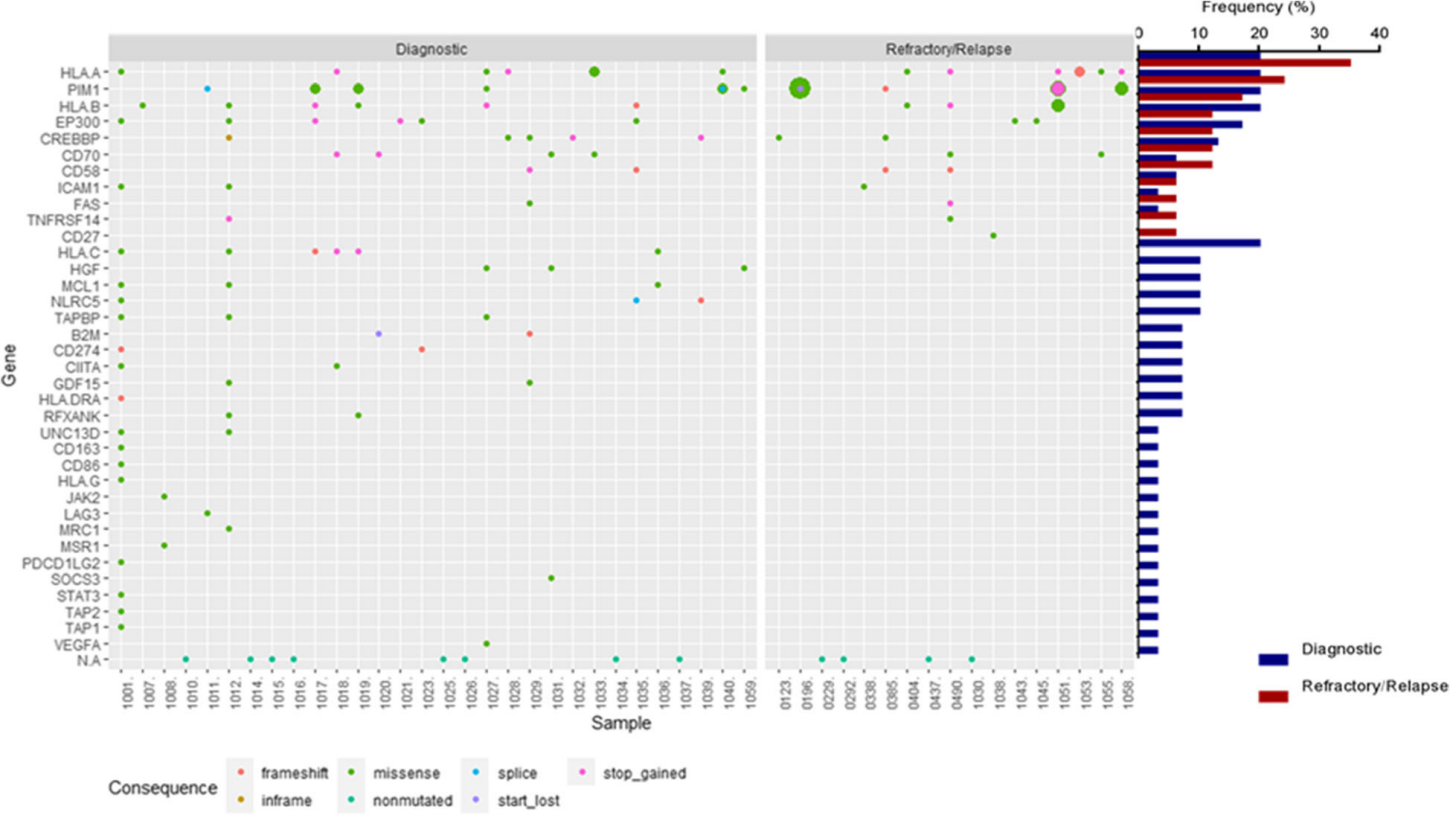
Mutational portrait of 36 immune surveillance genes in dDLBCL vs. rrDLBCL. The figure’s left side presents the immune surveillance genes in DLBCL, with gene mutation frequencies depicted on the right side calculated by including patients that do not harbor mutations in immune surveillance genes. Genes are sorted by the most frequently mutated genes in rrDLBCL. Samples that have circles on the last N. A row are samples that are not muatated by immune surveillance related genes. The circles’ size represents the number of specific mutations, while different colors present types of mutations. No individual genes are significantly different in gene mutation frequency between dDLBCLs and rrDLBCL tested by Fisher’s exact test. Also, we did not detect mutations in PDL1 and CTLA-4 genes

**Fig. 2:**
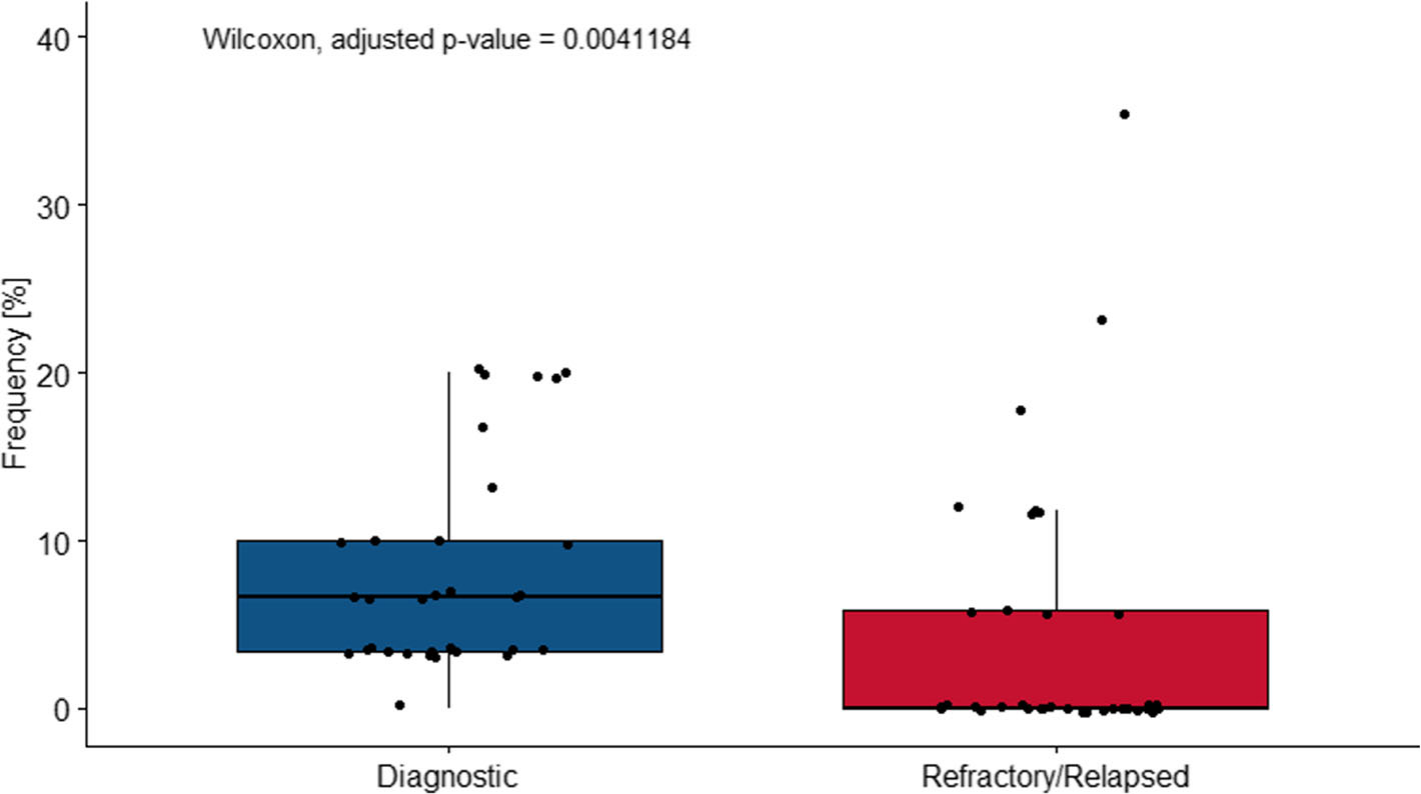
Gene mutation frequency for 36 immune surveillance genes differs between diagnostic (35 affected genes) and refractory/relapsed (11 affected genes) DLBCL patients. The Wilcoxon rank-sum test obtained the adjusted *p*-value

Genetic alterations in at least one of the 36 immune surveillance genes were detected in 22 (73%) dDLBCL and 13 (77%) rrDLBCL patients. The number of genetic alterations in dDLBCLs and rrDLBCLs ranged from 1 to 19 and 1–12 per patient, respectively (Fig. 1). Most of the detected variants in both dDLBCL and rrDLBCL were missense mutations followed by nonsense and frameshifts. No difference in the distribution of mutation types was observed for either dDLBCL or rrDLBCL (Fig. 1). Gene mutation frequencies in the 36 immune surveillance genes ranged from 3 to 20% and 6–35% in dDLBCLs and rrDLBCLs, respectively (Fig. 1 and S1), with significantly higher median gene mutation frequency in dDLBCLs (adjusted *p*-value = 0.002176, Wilcoxon rank-sum test, Fig. 2), affecting 3.2 fold more genes in dDLBCL (*n* = 35) than in rrDLBCL (*n* = 11). Thus, somatic variants in 25 immune surveillance genes were observed only in diagnostic patients, while only variants in *CD27* were restricted to rrDLBCLs (Fig. 2). Higher gene mutation frequencies were observed in *HLA-A, PIM1, CD58, FAS*, and *TNFRSF14* in rrDLBCLs compared to dDLBCL, even if none were significant.

**Fig. 3:**
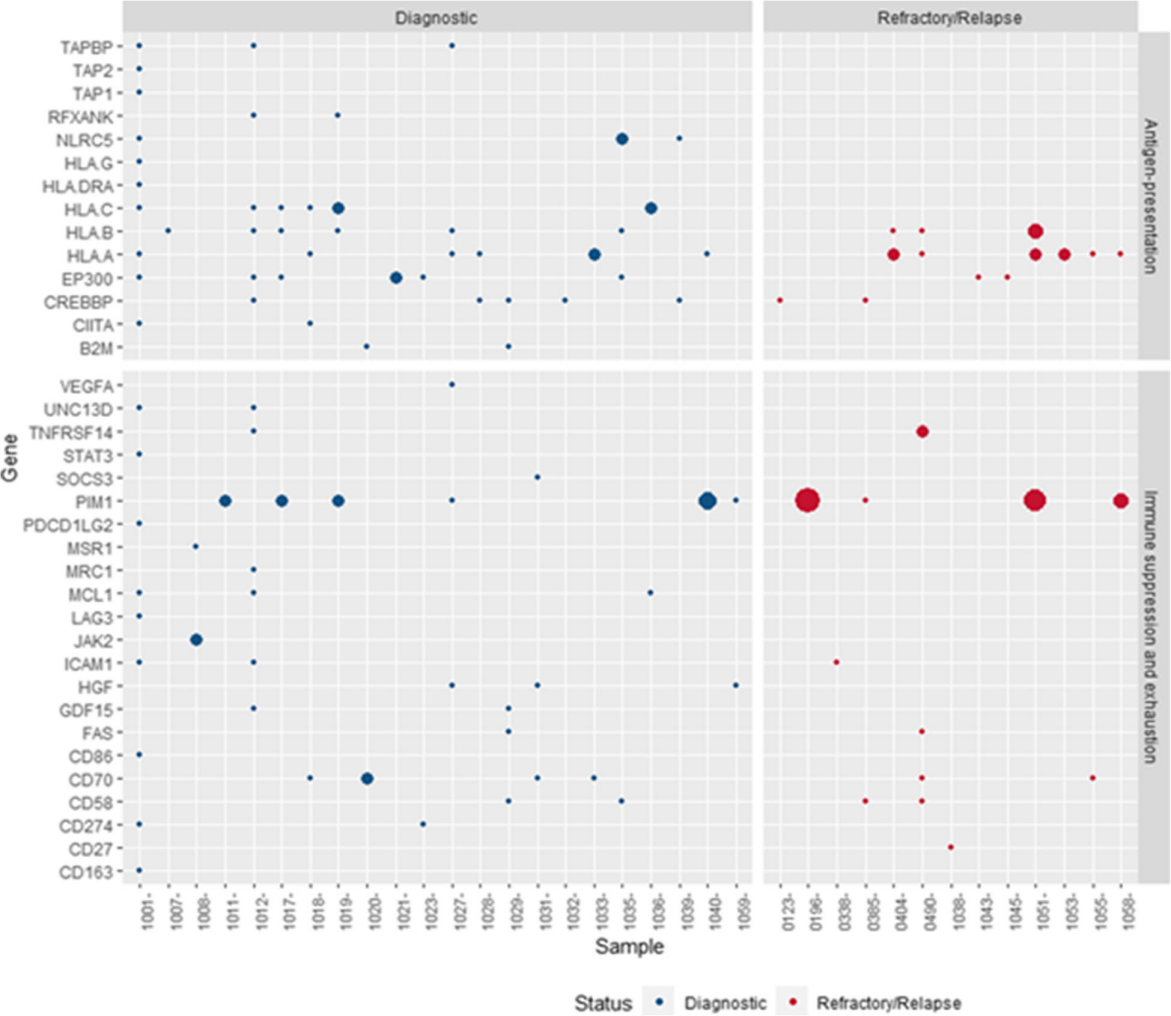
The mutational pattern of genes affecting antigene-presentation and immune suppression and exhaustion in dDLBCLs and rrDLBCLs. The blue dots represent mutations in diagnostic (dDLBCLs) patients, and red dots in refractory/relapsed patients (rrDLBCLs); the dots’ size represents the number of variants in a specific gene in the patient range (1–8). On the left side are genes representing sub group detected in our cohort. On the top are named cohorts

For 16% of dDLBCLs and 29% of rrDLBCLs genetic alterations were detected in genes involved in antigen presentation, while 13% of dDLBCLs and 12% of rrDLBCLs had genetic alterations in genes related to immune suppression and exhaustion (Fig. 3). The mutational patterns in targeted genes varied noteworthy, with few samples showing events in multiple genes or multiple events within one gene. Thus, diagnostic samples from 1007 and 1032 only harbored genetic events in one gene, *HLA-B* or *CREBBP*, respectively, whereas 19 genes were affected in sample 1001. For rrDLBCL, sample 0490 had six affected genes representing the maximum number of affected genes, whereas only one affected gene was observed for six other rrDLBCL samples (1053, 0123, 1038, 0338, 1045, 1043) (Fig. 3). Antigen-presenting genes were affected in more rrDLBCLs than dDLBCLs, and most of the patients in both cohorts harbored mutations in genes affecting both antigene presentation and immune suppression and exhaustion simultaneously 40 and 35% of dDBLCLs and rrDLBCLs, respectively. However, in dDLBCL, patients had mutations in antigen-presenting genes affecting both MHC-I and MHC-II, like sample 1028 with mutations in *CREBBP* and *HLA-A,* and 1039 with mutations in *CREBBP* and *NLRC5*, and patients who harbored mutations in genes affecting either MHC-I or MHC-II like sample 1007 and 1032, respectively. In contrast, rrDLBCLs harbored mutations in genes affecting either MHC-I (1053, 0404) or MHC-II (0123, 1045), which is also observed in our paired samples.

**Fig. 4:**
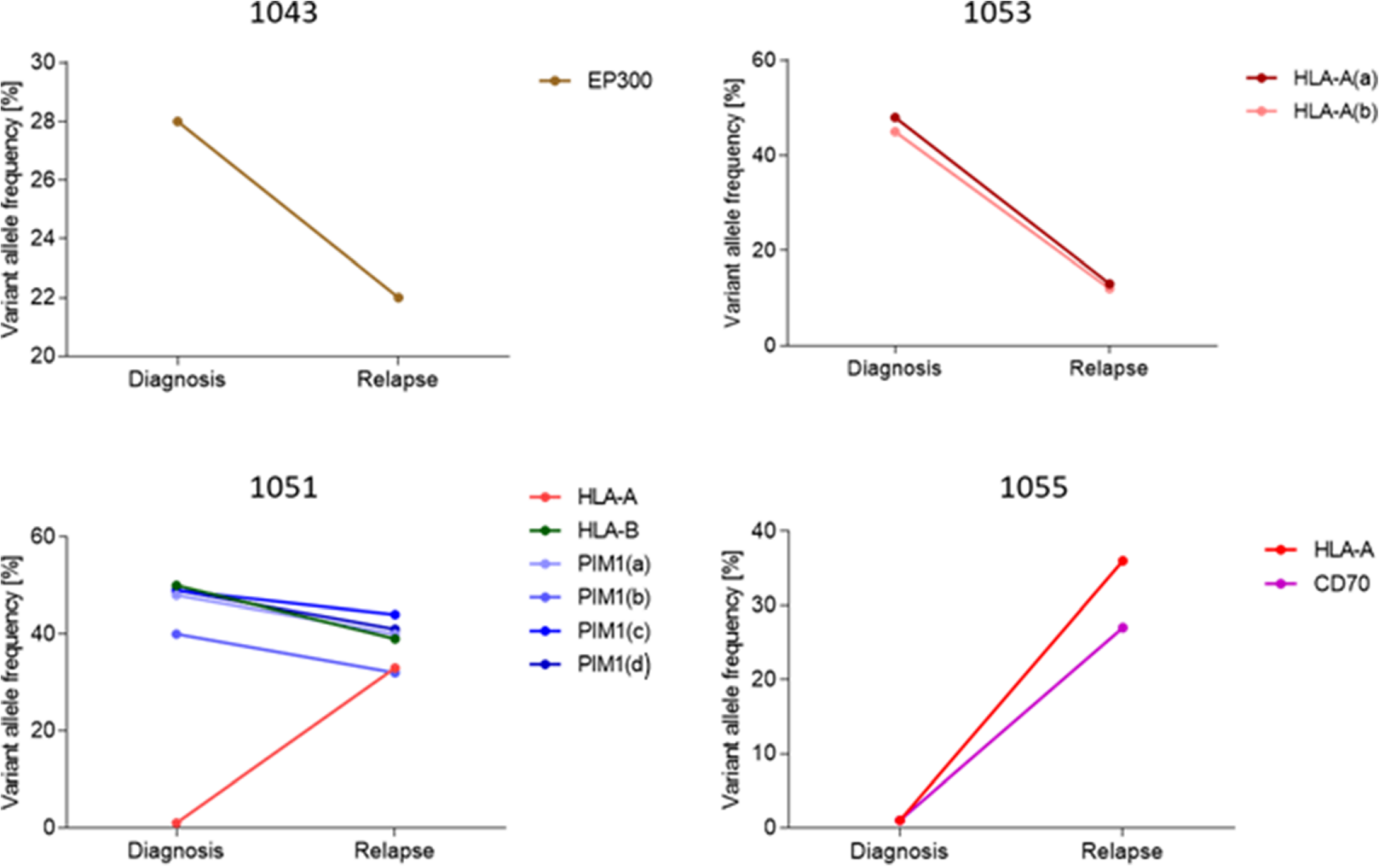
Exploration of genetic mutations in matched diagnostic and relapsed tumor biopsies of DLBCL patients. Variant allele frequency is displayed with the line as a percentage of each somatic coding mutation before and after treatment, showing possible clonal expansion; genes with additional letters (a-d) in brackets depict different mutations in the same genes

In four patients, we had pre-treatment biopsies collected at the time of diagnosis and post-treatment biopsy collected at the time of relapse or progression. Three patients were treated with R-CHOP (1043, 1053, and 1051), obtained complete remission, and relapsed after 0.8 years (median), with a median age of 74. Patient 1055 was treated with R-CHOEP, experienced progressive disease, and was biopsied after 0.7 years and at the age of 57 years. Variant allele frequencies (VAFs) of different individual mutations in affected genes differ between diagnostic and relapsed biopsies (Fig. 4), with all VAFs but *HLA-A* in patient 1051 being decreased in relapsed biopsies compared to diagnostic biopsies. Of notice, 1055 with progressive disease, genetic alterations in immune surveillance gens were not detected at the time of diagnosis, while at time of progression (9 months after diagnosis), mutations in *HLA-A* and *CD70* were detected. All of the matched samples harbored genetic variations in antigene presenting genes.

In addition, differential gene expression and Cibersort analysis was performed of dDLBCLs and rrDLBCLs comparing within each cohort samples with and without immune surveillance affected genes and samples with and without and MHC affected samples as well as comparison between dDLBCLs and rrDLBCLs. No significant difference difference by adjusted *P*-value were detected between any of the immune status comparisons. The only significant difference is observed in the proportion of Macrophages (M1) between dDLBCLs and rrDLBCLs harboring mutations in immune surveillance genes and genes related to antigen presentation (Fig. S2 A).

### Validation of the findings

To ensure the presence of next-generation sequencing (NGS) detected mutations, selected genetic variants were validated by ddPCR, as presented in Supplementary Table S1. For *CD58, CREBBP,* and *TNFRSF14*, a higher fractional abundance of mutant alleles was documented by ddPCR than detected by WES (Supplementary Table S1, Fig. S3).

**Fig. 5:**
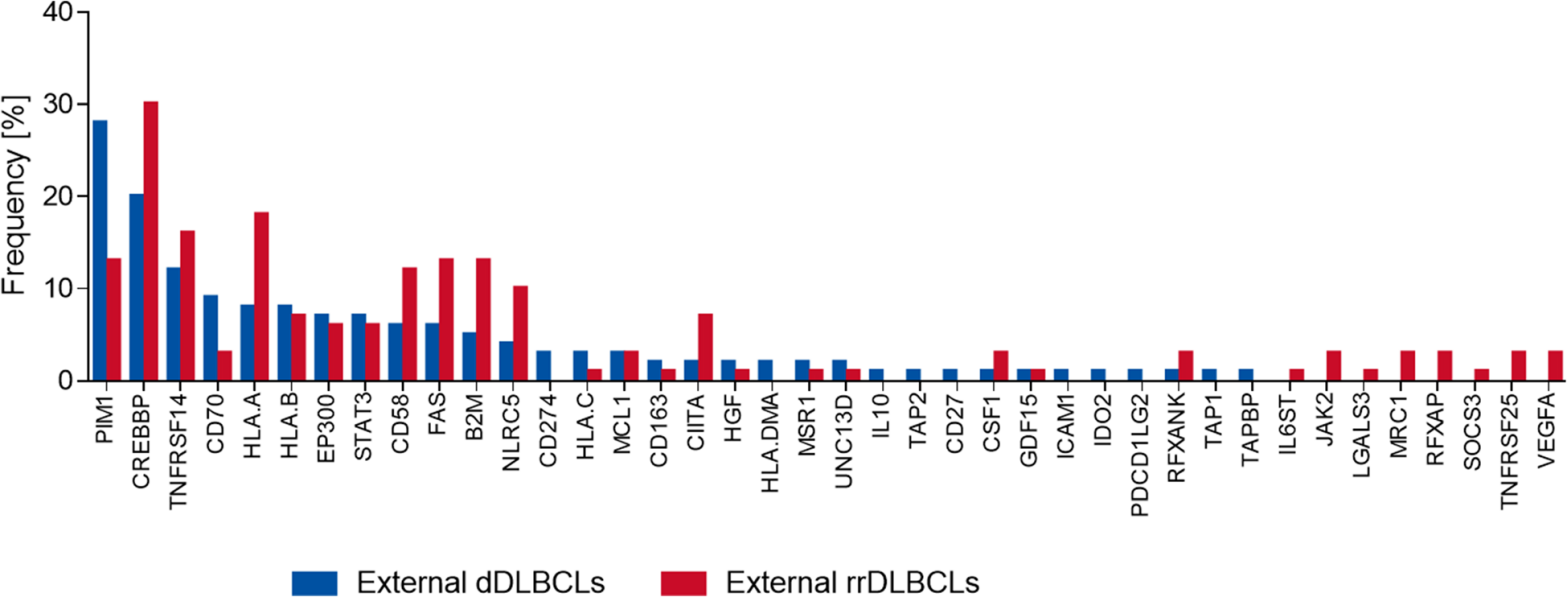
Gene mutation frequencies in the external diagnostic cohort (dDLBCLS) (Chapuy et al. *n* = 135) compared to merged refractory relapsed cohorts Morin et al. and Greenwalt et al. (*n* = 72) (rrDLBCLs)

Since our cohort was limited in size, we used publicly available WES datasets with sequenced tumor and matched germline samples, analyzing them in the same manner as our cohort to validate our observations on the mutational patterns of immune surveillance genes in DLBCL. Gene mutation frequencies in dDLBCL and rrDLBCL external cohorts were compared to evaluate if our detection of the gene mutation frequencies and observed prevalence of mutated antigene presenting genes were robust. The diagnostic cohort from Chapuy et al. (*n* = 135) was compared to merged refractory/relapsed cohorts by Morin et al. (*n* = 25) and Greenwalt et al. (*n* = 47) in order to obtain more refractory/relapsed samples. In the external dDLBCL and rrDLBCL comparable levels of samples (72 and 80%, respectively) harbored genetic variations in immune surveillance genes consistent with our observations. Gene mutation frequencies observed in the external cohorts were not significantly different between diagnostic and refractory/relapsed DLBCL samples as observed in our data (Fig. 5). Gene mutation frequencies in more than half (60%) of the mutated genes in external rrDLBCL were higher than in external dDLBCL even if the difference was not significant, which is in concordance with observations in our data (Fig. 5). Findings that differed between our study and the external cohorts were similar numbers of mutated immune surveillance genes in external rrDLBCLs (*n* = 30) and external dDLBCL (*n* = 32), demonstrating the important role of cohort size (Fig. S4).

## Discussion

We analyzed WES data to provide a genetic characterization of 58 curated immune surveillance genes in dDLBCL and rrDLBCL, post-treatment patients, which is essential for understanding the impact of the treatment in a genomic context. More than 70% of both dDLBCLs and rrDLBCLs patients harbor alterations in immune surveillance genes in our data, which is in concordance with external cohorts. No significant difference in individual gene mutation frequencies of immune surveillance genes between dDLBCLs and rrDLBCLs was observed in our data or in external cohorts, but in our rrDLBCL patients a smaller number of affected genes (*n* = 11) were observed than in dDLBCL (*n* = 35). A finding differing from external validation cohorts possibly introduced by diagnostic samples containing both cured and later relapsing patients, and in the rrDLBCL cohort, patients with both short and long time to new relapse could be included. However, more than half of genes affected in rrDLBCLs showed higher gene mutation frequency than in dDLBCLs, some near double (*HLA-A*) in our data and external cohorts (Figs. 1 and 5). Hence, during tumor progression and development of resistance in rrDLBCLs, we observed higher gene mutation frequencies in *PIM1, CD58, FAS, HLA-A*, and *TNFRSF14* as compared to dDLBCL consistent with other studies analyzing global genomic sequencing data [19, 27, 43] and illustrated the loss of immune surveillance (*CD58, HLA-A*) as well as immune suppression (*FAS, TNFRSF14, PIM1*) [19, 43]. Recognition and elimination by T- and NK- cells are avoided by mutated *CD58* and *HLA-A* genes [27, 44], which both have increased incidence in rrDLBCLs compared to dDLBCLS suggesting decreased immunogenicity of the tumor cells in the refractory and relapsed situation. In addition, observations from matched diagnostic and relapsed samples of the same patient (*n* = 3) depict decreased variant allele frequency in antigene presenting genes (e.g., *HLA-A)* but not vanishing after R-CHOP or R-CHOP-like treatment, suggesting a possible tumor evasion mechanism. This observation supports suggestion from Wise et al., 2020 that antigen presentation represents a key target for genetic alterations in rrDLBCL resistance [27]. The loss of function mutations in *FAS* gene lead to the suppression of Fas/FasL system responsible for activation-induced cell death [45]. In contrast, the loss of function mutations in the *TNFRSF14* gene leads to B-cell autonomous activation as well as extrinsic activation of the lymphoma microenvironment through B and T-lymphocyte attenuator (BTLA attenuator) located on CD4+ T-helper cells [46]. Specific missense mutations in *PIM1* are possibly activating. Along with PIM1 being overexpressed in DLBCL cells compared to normal B-cells, tumor cells are prevented from undergoing apoptosis inactivating proteins such as apoptosis signaling kinase 1 (ASK1), preventing further activation of FAS ligand [47, 48]. However, neoantigen presentation is necessary for immune surveillance, and lack of expression of MHC molecules might be the reason for failed anti-PD1 immunotherapies [10, 49]. In particular, if the cell is unable to present neoantigens in association with MHC molecules, there is no need for PD1/PD-L1 interaction [10]. Therefore, these patients might be good candidates for other immune therapies such as CAR-T-cell therapy, which has shown long-term response in approximately 58% of rrDLBCLs [27, 50], or bispecific CAR-T cell therapy that has approximately 80% overall response rate if patients receive freshly produced anti-CD19/20 [51]. Nevertheless, it is interesting to speculate that clonal selection of genetic variants in antigen-presenting genes occurs during or after the treatment resulting in the development of rrDLBCL (Fig. 4) even if we cannot distinguish single cell double genetic events from polyclonal tumor formations.

Recently, several algorithms have been developed, providing a refined classification of DLBCL into five to seven distinct subtypes based on genetic features [24, 52, 53]. As these genetic classes are based on global genetic analysis, and we use only a sub-selected set of genes in our analysis, we did not include refined genetic classification. However, it is observed that 73% of MCD genomes acquired genetic variants in genes affecting immune surveillance, thus becoming invisible to the host immune system, suggesting a crucial role in DLBCL pathogenesis, which is in agreement with cluster 5 described by Chapuy et al.,2018 [24, 40].

Also, an important observation in our dDLBCLs and rrDLBCLs is that 16 and 29% of the patients, respectively, harbor mutations in antigene presenting genes excluding genes in immune suppression and exhaustion, while 13 and 12% harbor mutations in genes related to immune suppression and exhaustion but not in antigen- presentation, respectively (Table S2). Similar features are observed in external dDLBCL and rrDLBCL cohorts where 15 and 28% of patients, respectively, are affected by mutated antigen-presenting genes and 25 and 25% affected by genes involved in immune suppression and exhaustion, respectively. Thus, a higher mutational rate of antigene presenting genes in rrDLBCL than in dDLBCL can be observed, though findings are not significant in neither our nor external cohorts – perhaps due to small cohort sizes it may suggest their possible role in the development of resistance toward therapy. Additionally, decreased proportion of M1 in rrDLBCLs may suggest a less aggressive host immune response by M1 in rrDLBCLs compared to dDLBCLS, but not restricted to rrDLBCLs harboring genetic alterations in immune surveillance or MHC related genes since no difference was observed in M1 proportions between rrDLBCLs with and without mutated immune surveillance or MHC associated genes. Also, no significant difference was observed in paired samples suggesting that the host does not respond or detect a difference in the tumor even if the IS genes are mutated and become invisible. Due to the limited number of paired samples, this observation requires more samples and tumor microenvironment analysis for validation.

## Conclusion

The role of genetic variations in the prevention of antigen presentation associated with MHC molecules and tumor suppression molecules is intriguing but offers new biological considerations encompassed in future investigations of larger cohorts of rrDLBCL and, ideally, the examination of paired diagnostic and relapse samples. Thus, successful treatment strategies for DLBCL may be to target multiple immune escape pathways simultaneously in a combination with more conventional treatments such as chemotherapy.

## Supplementary Information

**Additional file 1 Fig. S1.** Correlation of gene mutation frequency in diagnostic versus refractory/relapsed samples. Dots are representing the gene named above or pointed with the lines. A grey area represents the 95% confidence of the best-fit curve found by linear regression analyses. **Fig. S2.** The difference in a proportion of cell content: A – in dDLBCLS vs. rrDLBCLs with affected immune surveillance genes; B - in dDLBCLS vs. rrDLBCLs with affected MHC genes. **Fig. S3.** Validation of genetic variants by ddPCR in *TNFRSF14, CD58, CREBBP* gene. Allele frequency was detected in the same patients by NGS and ddPCR. **Fig. S4.** Distribution of mutations in external diagnostic (Chapuy et al.) and refractory/relapsed (Morin et al. and Greenwalt et al.) cohorts.

## Data Availability

The datasets used and/or analysed during the current study available from the corresponding author on reasonable request.

## Abbreviations

ABC: activated B-cell-like
ddPCR: digital droplet polymerase chain reaction
CAR-T: chimeric antigen receptor-T-cell
DLBCL: diffuse large B-cell lymphoma
GCB: the germinal center B-cell-like
dDLBCLs: diagnostic DLBCLs
IS: Immune surveillance
rrDLBCLs: refractory/relapsed DLBCLs
MHC-I`: major histocompatibility complex class I
MHC- II: major histocompatibility complex class II
M1: Macrophage 1; refractory/relapsed DLBCLs
R-CHOP: rituximab, cyclophosphamide, doxorubicin, vincristine, prednisone
NGS: next-generation sequencing
VAFs: Variant allele frequencies
