# Supplementary material for: The mutational profile of immune surveillance genes in diagnostic and refractory/relapsed DLBCLs
Source: BMC Cancer. 2021 Jul 18;21:829. doi: 10.1186/s12885-021-08556-3 (PMC8286604; doi:10.1186/s12885-021-08556-3)

Supplementary document S1

**The mutational profile of immune surveillance genes in diagnostic and refractory/relapsed DLBCLs**

Marijana Nesic, Mads Sønderkær, Rasmus Froberg Brøndum^,^_,_ Tarec Christoffer El-Galaly, Inge Søkilde Pedersen_,_ Martin Bøgsted, Karen Dybkær

**Supplementary Tables**


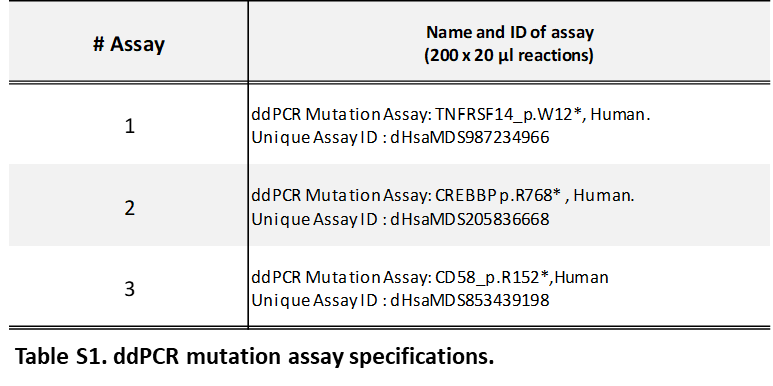


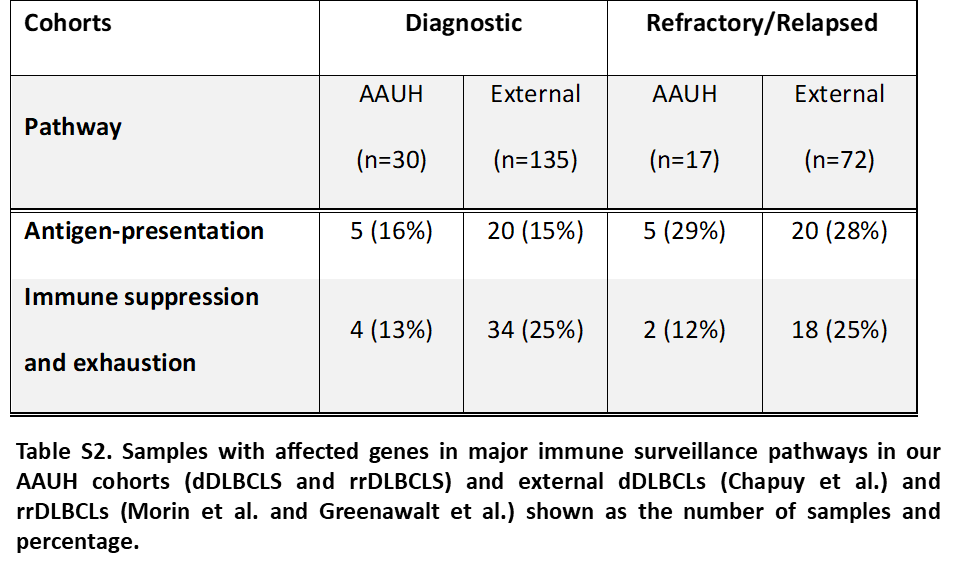


**Supplementary Figures**


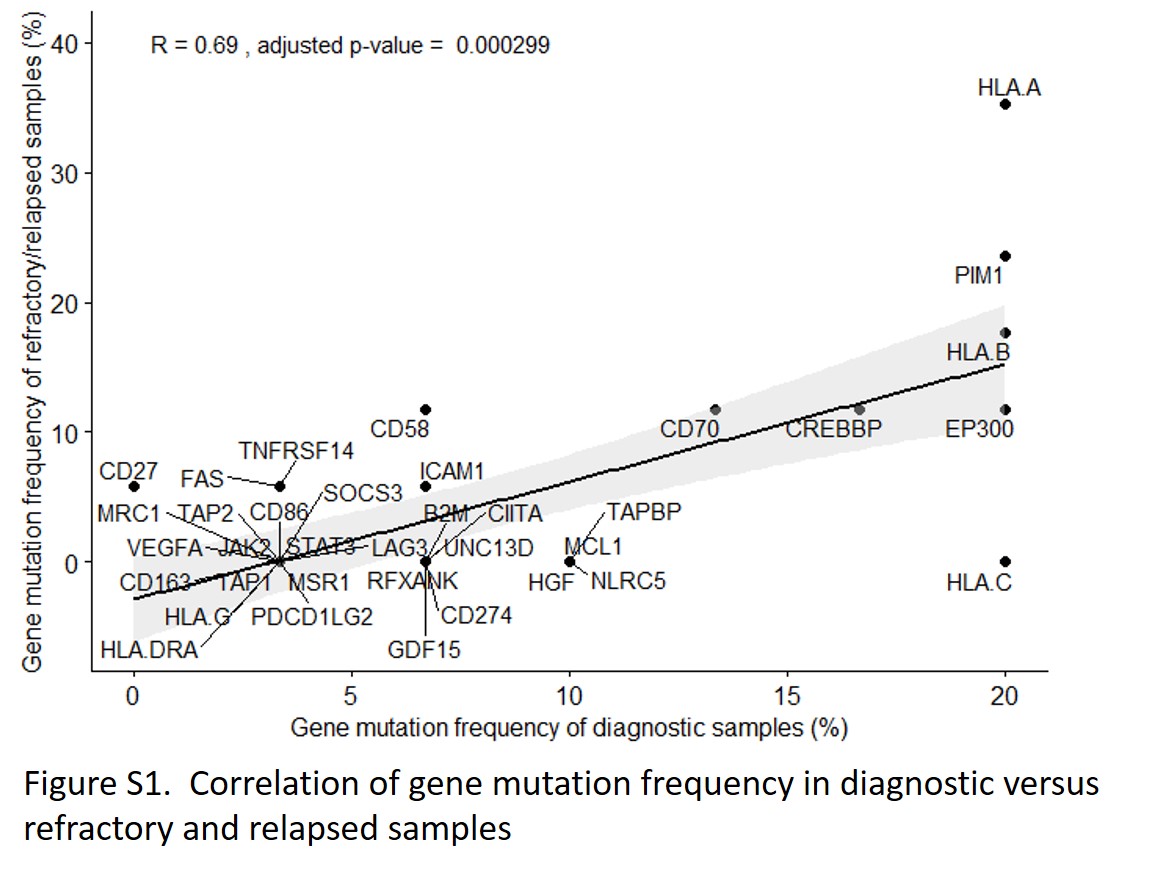


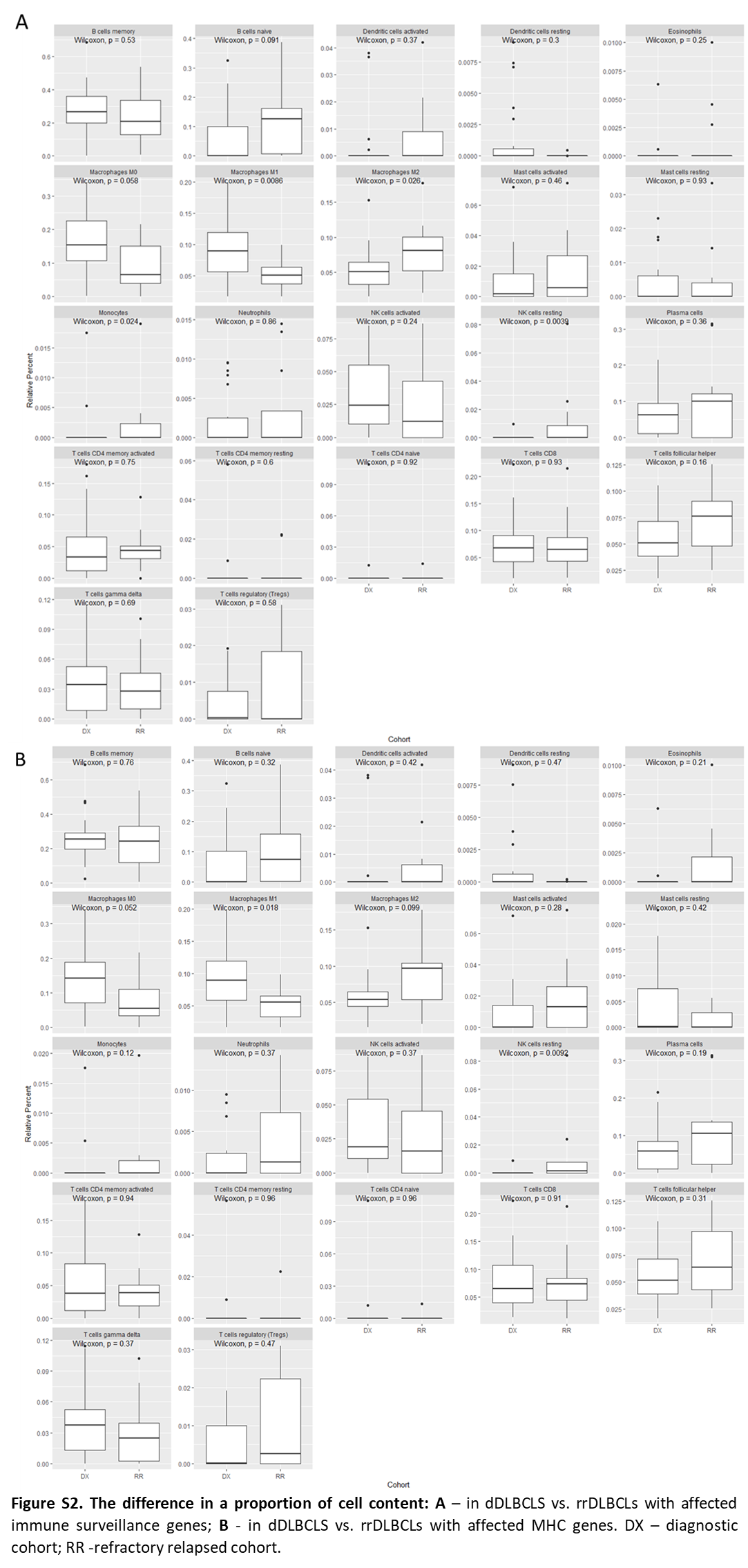


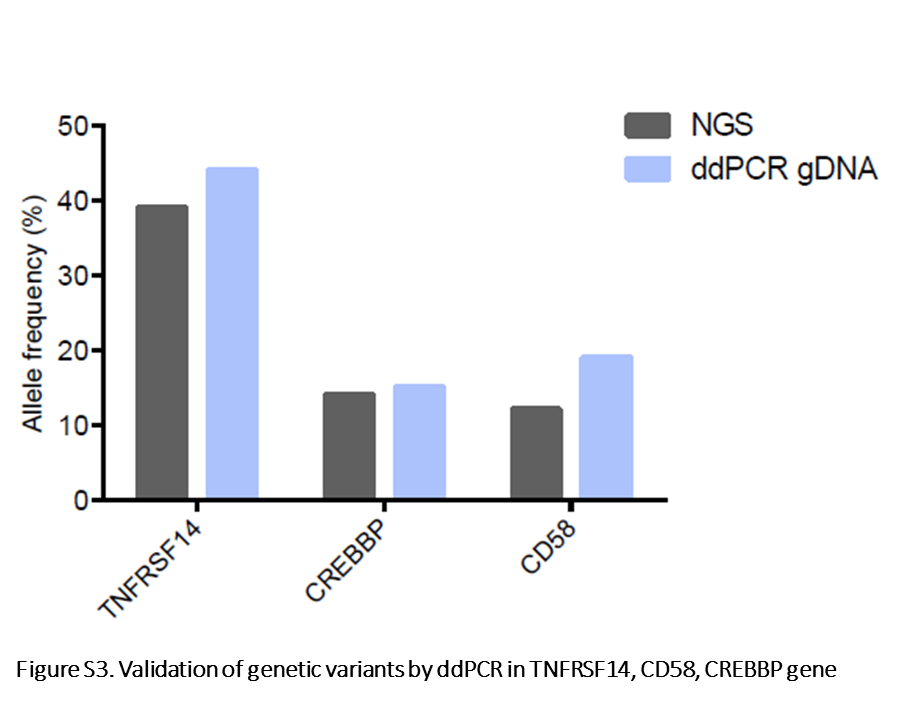


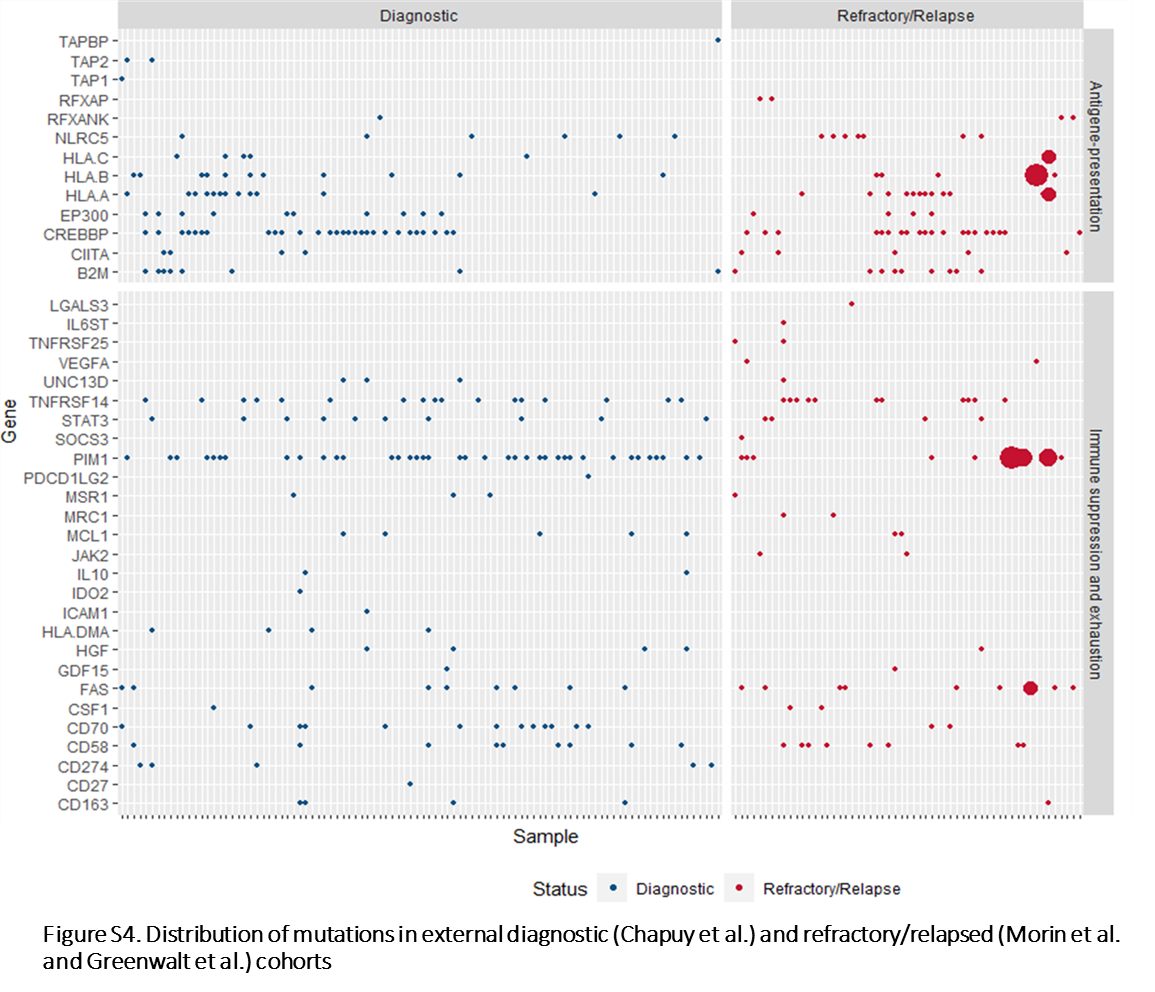

Supplement: Supplementary file 1 — Additional file 1 Fig. S1. Correlation of gene mutation frequency in diagnostic versus refractory/relapsed samples. Dots are representing the gene named above or pointed with the lines. A grey area represents the 95% confidence of the best-fit curve found by linear regression analyses. Fig. S2. The difference in a proportion of cell content: A – in dDLBCLS vs. rrDLBCLs with affected immune surveillance genes; B - in dDLBCLS vs. rrDLBCLs with affected MHC genes. Fig. S3. Validation of genetic variants by ddPCR in TNFRSF14, CD58, CREBBP gene. Allele frequency was detected in the same patients by NGS and ddPCR. Fig. S4. Distribution of mutations in external diagnostic (Chapuy et al.) and refractory/relapsed (Morin et al. and Greenwalt et al.) cohorts. [file 12885_2021_8556_MOESM1_ESM.docx]
